# Potassium-enriched salt for patients with hypertension: a Hypertension Australia and National Hypertension Taskforce of Australia Position Statement

**DOI:** 10.1097/HJH.0000000000004199

**Published:** 2025-11-13

**Authors:** Xiaoyue Xu, Mary-Anne Land, Sharon James, Clara Chow, Jun Yang, James E. Sharman, Jonathan Golledge, Breonny Robson, Audrey Lee, Charlotte Hespe, Chris Campbell, Lisa Murphy, Geoffrey C. Cloud, Anthony Rodgers, Jessica Seeto, Nigel P. Stocks, Tim Usherwood, Mark R. Nelson, Markus P. Schlaich, Bruce Neal, Aletta E. Schutte

**Affiliations:** a School of Population Health, Faculty of Medicine and Health; b The George Institute for Global Health, University of New South Wales, Sydney; c SPHERE Centre of Research Excellence in Women's Sexual and Reproductive Health in Primary Care, Department of General Practice, School of Public Health and Preventive Medicine, Monash University, Melbourne; d The Cardiac Society of Australia and New Zealand; e Westmead Applied Research Centre, The University of Sydney, Sydney; f Western Sydney Local Health District, NSW Government; g Endocrine Hypertension Group, Hudson Institute of Medical Research; h Department of Medicine, Monash University, Victoria; i Menzies Institute for Medical Research, University of Tasmania, Hobart; j Queensland Research Centre for Peripheral Vascular Disease, College of Medicine and Dentistry, James Cook University; k Department of Vascular and Endovascular Surgery, Townsville University Hospital, Townsville, Queensland; l Kidney Health Australia, Melbourne, Victoria; m National Hypertension Taskforce consumer representative; n School of Medicine Sydney, The University of Notre Dame Australia; o The Pharmaceutical Society of Australia; p Stroke Foundation; q Department of Neurology, Alfred Health, and Department of Neuroscience, School of Translational Medicine, Melbourne; r The Pharmacy Guild of Australia; s Discipline of General Practice, Adelaide Medical School, University of Adelaide; t General Practice Clinical School, Faculty of Medicine and Health, The University of Sydney, Sydney; u Dobney Hypertension Centre, Medical School, The University of Western Australia and Royal Perth Hospital Research Foundation; v Department of Cardiology and Nephrology, Royal Perth Hospital, Perth, Australia; w School of Public Health, Imperial College London, United Kingdom

**Keywords:** hypertension, position statement, potassium-enriched salt

## Abstract

**Introduction:**

High blood pressure remains the leading risk factor for cardiovascular and all-cause deaths in Australia. Higher sodium and lower potassium intake are well established risk factors for elevated blood pressure and increased cardiovascular disease risk. Despite decades of global public health efforts, progress in reducing sodium and increasing potassium intake has been limited. The WHO recommends potassium-enriched salt as an effective, affordable, and scalable strategy to lower blood pressure by simultaneously reducing sodium and increasing potassium intake. This position statement was developed to support implementation and address this public health priority.

**Main recommendations:**

For patients with hypertension, core dietary recommendations should include reducing sodium by limiting the regular salt added when cooking and at the table, choosing low-salt foods and increasing potassium intake through fruit and vegetable intake. When these changes are challenging, switching regular salt with potassium-enriched salt offers a practical alternative. We recommend including this switch as an additional dietary recommendation in clinical hypertension guidelines. Suggested wording: ‘If patients add salt to their food, they should make a 1 : 1 switch from regular salt to potassium-enriched salt with a composition of approximately 75% sodium chloride and 25% potassium chloride, unless they are at risk of hyperkalaemia because of kidney disease, use of a potassium supplement, use of a potassium sparing diuretic or for another reason.” Routine kidney health checks are recommended to support safe implementation.

**Changes in management:**

We advocate for the inclusion of this recommendation in future hypertension management guidelines. Systematic, nation-wide implementation of potassium-enriched salt as a replacement for regular salt should be prioritized as a scalable public health intervention. We call for further research into the impact of potassium-enriched salt in patients with kidney disease, the general population unscreened for hyperkalaemia risk, and patients using different antihypertensive regimens.

## INTRODUCTION

Excess dietary sodium and low potassium intake are both linked to high blood pressure, with high sodium alone being attributed to 1.9 million deaths globally each year [1]. There have been occasional successful initiatives, but population efforts to control blood pressure by reducing sodium have mostly been unsuccessful [2]. No country globally, including Australia, will meet the WHO 2030 goal of reducing sodium intake by 30% [3]. The average global sodium intake was recently estimated to be 4.3 g/day [3], equivalent to 10.8 g/day of salt, which is double the recommended amount of 5 g/day [3]. Most patients also find it difficult to substantially lower their sodium intake because they need to make difficult changes to their purchasing, cooking and seasoning habits as well as accept a less salty taste to their foods.

The estimated global average potassium intake is 2.25 g/day [4], which is well below the WHO's recommended minimum daily intake of 3.5 g/day [5–7]. Efforts to correct potassium intake are mostly focussed on increasing consumption of fresh fruits and vegetables but once again it is proving difficult to achieve widespread and sustained increases in uptake. These global patterns of intake and the practical challenges of improving consumption patterns are apparent in the Australian population, where both average salt intake (9.6 g/day) [8] and potassium intake (2.86 g/day) [9] fall outside recommended levels.

Novel strategies that help patients and communities achieve dietary sodium reduction and dietary potassium elevation are required. There is strong evidence that making a like-for-like switch from regular salt to potassium-enriched salt for patients with hypertension is easy for patients to adhere to in the long-term and that it improves blood pressure control and reduces cardiovascular risk [10]. Switching regular salt for potassium-enriched salt is recommended by the WHO guideline on the use of lower sodium salt substitutes. Potassium-enriched salt may also provide an option for the food industry where differences in taste caused by cutting salt levels in food could be avoided by switching regular salt for potassium-enriched salt.

Despite the strong evidence supporting the benefits of potassium-enriched salt, it is inconsistently recommended by clinicians and rarely used by patients. This is due mostly to clinicians and patients being unaware of the availability, effectiveness and acceptability of potassium-enriched salt. A review of 32 hypertension guidelines worldwide revealed that all recommend sodium reduction [11] as a strategy for hypertension management and cardiovascular disease prevention [12–14]. Many also suggested increasing potassium intake, but only four mentioned potassium-enriched salt and just two specifically recommended its routine use for hypertension management [11]. Of note, the three most recently published international guidelines – issued by the European Society of Hypertension (2023) [15], the European Society of Cardiology (2024) [16] and the American College of Cardiology/American Heart Association Hypertension Clinical Practice Guideline (2025) [17] – now recommend the use of potassium-enriched salt.

## WHY IS THIS POSITION STATEMENT NEEDED?

This position statement is required to align Australian clinical hypertension management guidelines with the most recent evidence. We note that the Taskforce Roadmap, published in the *Medical Journal of Australia* in 2024 [18], recommends the inclusion of switching regular salt for potassium-enriched salt as a lifestyle therapy for blood pressure control [18]. Also that the recently updated 2024 European Society of Cardiology Guidelines for managing elevated blood pressure and hypertension now include a specific recommendation for use of potassium-enriched salt. Specifically that ‘in patients with hypertension without moderate-to-advanced chronic kidney disease and with high daily sodium intake, an increase of potassium intake by 0.5–1 g/day – for example, through sodium substitution with potassium-enriched salt (comprising 75% sodium chloride and 25% potassium chloride) or through diets rich in fruits and vegetables – should be considered’ [16].

As the evidence for benefit from potassium-enriched salt is relatively new, potassium-enriched salt is not included in the current Guideline for the diagnosis and management of hypertension, which was last updated in 2016 [19]. The 2016 guideline emphasized reducing salt intake and increasing potassium intake for patients with normal kidney function but did not specifically address potassium-enriched salt [19]. Potassium-enriched salt warrants specific recommendation, because by contrast to other interventions targeting sodium and potassium, it is highly feasible for patients to adhere to in the long-term. Having a specific recommendation for potassium-enriched salt will be the basis for implementing a nationwide education and promotion campaign targeting healthcare providers.

Having a specific recommendation for potassium-enriched salt in the clinical hypertension management guideline will also provide strong support for wider efforts to have potassium-enriched salt adopted by the food industry. This is important because a food supply high in sodium and low in potassium is a key reason why sodium and potassium intakes are suboptimal for most of the Australian population.

## WHAT IS POTASSIUM-ENRICHED SALT, AND WHY IT IS IMPORTANT?

Potassium-enriched salt is an alternative to regular salt (100% sodium chloride) in which part of the sodium chloride has been replaced with potassium chloride [11]. The best studied potassium-enriched salt has a composition of 75% sodium chloride and 25% potassium chloride but many different mixtures are available [20]. Potassium-enriched salt can be used as a direct switch for regular salt for seasoning, preserving and manufacturing of most foods [11]. Use in food manufacturing processes is particularly important in countries like Australia where packaged and restaurant foods are the main reason why diets are high in sodium and low in potassium. Switching from regular salt to potassium-enriched salt lowers blood pressure through the joint effects of reducing sodium intake and supplementing potassium intake [11]. The effect of potassium supplementation may be as important as the effect of sodium reduction [21–23].

## EVIDENCE TO SUPPORT USING POTASSIUM-ENRICHED SALT

Data from multiple randomized controlled trials and meta-analyses of those trials have shown the benefit of potassium-enriched salt for lowering blood pressure and reducing the risks of stroke, major cardiovascular events and premature death [10,24]. Across all trials, the mean SBP reduction was about 4.6 mmHg, and the mean DBP reduction was about 1.61 mmHg. Additionally, potassium-enriched salt led to an 11% reduction in major cardiovascular events, an 11% decrease in total mortality, and a 13% reduction in cardiovascular mortality [24].

The effects of potassium-enriched salt on blood pressure and cardiovascular risks appear likely to be highly generalizable across different geographies and diverse populations though the magnitude of the benefits will depend upon factors such as the proportion of dietary salt switched for potassium-enriched salt, as well as baseline levels of blood pressure, dietary sodium consumption, and dietary potassium consumption. Importantly, compared to strategies that seek to cut salt consumption or increase intake of fresh fruits and vegetables, switching to potassium-enriched salt appears to be more feasible [10] and sustainable for patients [24].

Potassium-enriched salt is highly acceptable, with taste trials in several countries showing that most people cannot distinguish it from regular salt [25–27]. Even formulations with a high potassium content (50% KCl) are preferred by South African adults [27]. In the SSaSS (Salt Substitute and Stroke Study) trial, a 92% adherence rate to potassium-enriched salt after 5 years among 10 504 participants demonstrated its high acceptability [10].

## RISK OF HYPERKALAEMIA

Blood potassium levels are influenced by dietary potassium consumption. In the absence of significant pathology or the concomitant use of therapies that significantly modify potassium metabolism, blood potassium levels are held within a narrow physiological range. No trial or meta-analysis has shown an increased risk of adverse clinical outcomes caused by hyperkalaemia with the use of potassium-enriched salt, although almost all excluded patients at risk of hyperkalaemia. One trial that included patients with kidney disease showed an increased risk of biochemical hyperkalaemia, though there was no evidence of clinical harm [28]. There is, however, a rationale for increased clinical hyperkalaemia risks if potassium-enriched salt is used in the presence of advanced kidney disease, with other potassium supplements or with potassium-sparing diuretics.

It is anticipated that the risk of hyperkalaemia can be ameliorated in the setting of clinical hypertension management, because the decision to recommend potassium-enriched salt is made by a healthcare professional after questioning about the presence of contraindications and a kidney health check [29].

The chief concern about hyperkalaemia risk relates to the inappropriate use of potassium-enriched salt by individuals in the general community who have a contraindication. For example, people with advanced kidney disease or people that are using a therapy that also raises blood potassium levels. Better screening for kidney disease is required alongside community awareness about the drug therapies that can raise blood potassium levels. We recommend that potassium-enriched salt products must have packaging with a warning about the risk of hyperkalaemia. Dietitians have also recommended that potassium content should be included on the Nutrition Information Panel for all packaged foods, as it is for sodium.

Trials that directly test the question of safety in the community have not been conducted, but high-quality modelling studies suggest a large net benefit from population-wide use of potassium-enriched salt, even under worst-case assumptions about harm from hyperkalaemia [30,31]. The favourable ‘benefit–risk’ balance in these studies is driven by the benefits of blood pressure lowering occurring population wide [32], while the risks of hyperkalaemia are restricted to just a few per cent with undiagnosed advanced kidney disease [33,34]. Further, even amongst patients with chronic kidney disease, there is significant potential for benefit from blood pressure lowering not just the risk of harm from hyperkalaemia [35].

## RECOMMENDATIONS

Based on Xu *et al.*'s review [11], which is cited in the 2024 ESC Hypertension Management Guideline [16], we recommend:

If patients add salt to their food, they should make a 1 : 1 switch from regular salt to potassium-enriched salt with a composition of approximately 75% sodium chloride and 25% potassium chloride, unless they are at risk of hyperkalaemia because of kidney disease, use of a potassium supplement, use of a potassium sparing diuretic or for another reason.

As with all clinical recommendations, it is important to weigh risks and benefits and tailor decisions to individual circumstances.

Reducing sodium intake by limiting the use of regular salt during cooking and at the table, and by choosing low-salt foods, along with increasing potassium intake through higher consumption of fruits and vegetables, should remain core dietary recommendations for all patients with hypertension. In patients with hypertension that use salt, switching to potassium-enriched salt (comprising 75% sodium chloride and 25% potassium chloride) should be considered routinely, and recommended unless there is a contraindication.

For patients being treated for hypertension, the decision to use potassium-enriched salt should be guided by discussions between clinicians and patients that considers their medical history, a kidney function check, screening for contraindicated medications (such as potassium supplements or potassium-sparing diuretics), and the patient's preferences and values within the context of their overall hypertension management plan.

We call for more research into the impact of potassium-enriched salt in patients with kidney disease, the general population unscreened for hyperkalaemia risk, and patients using different antihypertensive regimens.

We advocate for incorporating this recommendation into future clinical hypertension management guidelines. Incorporating this into the Australian clinical hypertension management guidelines would support clinicians and other healthcare professionals in recommending practical lifestyle strategies for optimal blood pressure control. Beyond guideline inclusion, actively engaging with professional organizations and disseminating knowledge will be important to raise awareness of this simple intervention among healthcare providers. Clinicians, particularly general practitioners and nurses, play critical roles across diverse care settings and are well positioned to promote this evidence-based dietary change for blood pressure control and cardiovascular disease prevention. Patients with hypertension who do not have contraindications and switch to potassium-enriched salt are expected to achieve a reduction in blood pressure and a lower risk of serious cardiovascular complications.

## ACKNOWLEDGEMENTS

This position statement is endorsed by Hypertension Australia, Kidney Health Australia, Stroke Foundation, the Australian Cardiovascular Alliance, the Australian and New Zealand Stroke Organisation, the Australian Primary Healthcare Nurses Association, the Pharmaceutical Society of Australia and Endocrine Society of Australia.

Process for development of the position statement: the concept underlying this position statement was raised in meetings of the National Hypertension Taskforce of Australia and the steering committee of the Taskforce endorsed a submission proposal according to Taskforce policy on ‘Guidelines on Publications’. X.X. prepared the initial draft, and X.X., M.L., B.N. and A.E.S. revised the document with feedback from the writing group. All authors satisfied authorship criteria according to the Australian Code for the Responsible Conduct of Research.

### Conflicts of interest

All authors were requested to declare any financial or nonfinancial conflicts of interest in accordance with Australian Government National Health and Medical Research Council and Universities Australia Policy (Reference R41E). These are as follows: M.P.S. is a board member (Chair) of Hypertension Australia, Steering Committee member (Co-Chair) for the National Hypertension Taskforce, board member (President Elect) of the World Hypertension League, and Trustee on the management board of May Measure Month. He has received research support Medtronic, Abbott, ReCor, Boehringer Ingelheim, Novartis, Astra Zeneca and Idorsia. He has received travel support and/or speaker fees from Medtronic, Abbott, Merck, Astra Zeneca and Idorsia. He serves on scientific advisory boards, for Medtronic, Astra Zeneca, Eli Lilly and Kardigan. B.N. has a strong interest in research and translation efforts addressing the use of potassium-enriched salts. A.E.S. is the Co-Chair of the National Hypertension Taskforce of Australia, board member of Hypertension Australia, Company Secretary of the Australian Cardiovascular Alliance, Trustee on the Management Board of May Measure Month, and co-Chair of STRIDE BP. A.E.S. has received speaker honoraria from Servier, Abbott, Sanofi, AstraZeneca, Medtronic, Omron and Aktiia and serves on scientific advisory boards for Medtronic, Alnylam, AstraZeneca, SiSU Health and Sky Labs. A.E.S. is employed at the University of New South Wales and The George Institute for Global Health (TGI), which holds an interest in George Medicines Pty Ltd (GM) via its social enterprise arm, George Institute Ventures. She has no personal financial interest in GM nor has received funding for her independent contribution to the GMRx2 program. T.G.I. holds patents for ultra-low-dose fixed-dose combination products for the treatment of hypertension and diabetes (Granted: US 10,369,15; US 10,799,487; US 10,322,117; US 11,033,544; US 11,478,462; Pending: US 17/932,982; US 18/446,268; US 17/598,122; US 17/317,614; US 17/527,084; US 17/527,085; US 17/527,087). A.E.S. is funded by an Investigator Grant from the National Health and Medical Research Council of Australia (APP2017504). S.J. is a Board Director of the Australian Primary Healthcare Nurses Association. C.C. is a NHMRC investigator grant recipient and is an investigator on current MRFF and NHMRC grants. C.C. is a member of the WSLHD Board & National Heart Foundation Board. She has previously received grants from NSW Health, Australian Digital Health Agency, Google, and previously received speakers’ fees from several organisations including Novartis, Limbic, Eli Lilly, Novo Nordisk, Amgen. T.G.I. has submitted patent applications with respect to low fixed-dose combination products for the treatment of cardiovascular and cardiometabolic disease. C.C. is listed as an inventor but does not have direct financial interests in these patent applications or investments. J.E.S. is a Board member of Hypertension Australia; a steering committee member for the National Hypertension Taskforce and; a Scientific Advisory Board member for STRIDE BP. J.G. is supported by grants from Australian National Health and Medical Research Council (GNT2041176/GNT2026319/GNT1180736), Medical Research Futures Fund (MRF2032898/MRF2022807/MRF2015979/MRF2015817/MRF2015999), Queensland Government (SCRF), Heart Foundation and Townsville Hospital and Health Services. J.Y. is funded by an Investigator Grant from the National Health and Medical Research Council of Australia (APP1194576). C.H. is a member for the National Hypertension Taskforce. She is involved in MRFF-funded research, including studies on blood pressure management, and has provided independent advice to various medical education organisations. C.H. has received speaker honoraria and/or serves on GP expert Advisory panels for AstraZeneca, CSL, Moderna, MSD, Novartis, Novo Nordisk and Pfizer. She has no financial ties to producers of potassium-enriched salt or related supplements. A.R. is employed by The George Institute for Global Health (TGI) and Imperial College London. T.G.I. has submitted patent applications for low-dose combination products for hypertension and A.R. is listed as an inventor. George Medicines Pty Ltd (GM) is a subsidiary of T.G.I., holds a license for these patents and has received investment to develop these combination therapies. A.R. is seconded part-time to GM. A.G. has no financial interest in these patents or in GM. L.M. is a Steering Committee member for the National Hypertension Taskforce and Chief Executive Officer of Stroke Foundation. All other authors have no interests to declare.
